# Immunogenic cell death-related gene landscape predicts the overall survival and immune infiltration status of ovarian cancer

**DOI:** 10.3389/fgene.2022.1001239

**Published:** 2022-11-08

**Authors:** Wenwen Zhang, Tianbo Liu, Liangliang Jiang, Jiarong Chen, Qiuli Li, Jing Wang

**Affiliations:** Department of Gynecology, Harbin Medical University Cancer Hospital, Harbin, Heilongjiang, China

**Keywords:** ovarian cancer, immunogenic cell death (ICD), risk model, prognosis, immune infiltration

## Abstract

**Background:** Ovarian cancer (OC) is the most troubling malignant tumor of the female reproductive system. It has a low early diagnosis rate and a high tumor recurrence rate after treatment. Immunogenic cell death (ICD) is a unique form of regulated cell death that can activate the adaptive immune system through the release of DAMPs and cytokines in immunocompromised hosts and establish long-term immunologic memory. Therefore, this study aims to explore the prognostic value and underlying mechanisms of ICD-related genes in OC on the basis of characteristics.

**Methods:** The gene expression profiles and related clinical information of OC were downloaded from The Cancer Genome Atlas (TCGA) and Gene Expression Omnibus (GEO) database. ICD-related genes were collected from the Genecards database. ICD-related prognostic genes were obtained by intersecting ICD-related genes with the OC prognostic-related genes that were analyzed in the TCGA database. Functional enrichment, genetic mutation, and immune infiltration correlation analyses were further performed to identify underlying mechanisms. Subsequently, we developed a TCGA cohort-based prognostic risk model that included a nine-gene signature through univariate and multivariate Cox regression and LASSO regression analyses. Meanwhile, external validation was performed on two sets of GEO cohorts and the TCGA training cohort for three other common tumors in women. In addition, a nomogram was established by integrating clinicopathological features and ICD-related gene signature to predict survival probability. Finally, functional enrichment and immune infiltration analyses were performed on the two risk subgroups.

**Results:** By utilizing nine genes (ERBB2, RB1, CCR7, CD38, IFNB1, ANXA2, CXCL9, SLC9A1, and SLAMF7), we constructed an ICD-related prognostic signature. Subsequently, patients were subdivided into high- and low-risk subgroups in accordance with the median value of the risk score. In multivariate Cox regression analyses, risk score was an independent prognostic factor (hazard ratio = 2.783; *p* < 0.01). In the TCGA training cohort and the two GEO validation cohorts, patients with high-risk scores had worse prognosis than those with low-risk scores (*p* < 0.05). The time-dependent receiver operating characteristic curve further validated the prognostic power of the gene signature. Finally, gene set enrichment analysis indicated that multiple oncological pathways were significantly enriched in the high-risk subgroup. By contrast, the low-risk subgroup was strongly related to the immune-related signaling pathways. Immune infiltration analysis further illustrated that most immune cells showed higher levels of infiltration in the low-risk subgroup than in the high-risk subgroup.

**Conclusion:** We constructed a novel ICD-related gene model for forecasting the prognosis and immune infiltration status of patients with OC. In the future, new ICD-related genes may provide novel potential targets for the therapeutic intervention of OC.

## 1 Introduction

Among all malignant diseases of the female reproductive system, ovarian cancer (OC) is one of the most troublesome. The most recent cancer statistics show that OC is expected to account for 19,880 new cases and 12,810 fatalities in the United States in 2022 (Siegel et al., 2022). OC has an incidence rate that ranks second among all gynecological tumor diseases (17.3%), but its mortality rate jumps to the first place (39.0%) mainly due to the following reasons: First, the ovaries are located deep in the pelvis, and the early stage of OC has almost no symptoms. Therefore, most patients are already in the advanced stage when they are diagnosed with OC (Wu et al., 2022). Second, the effectiveness of initial treatment in patients with OC has been limited due to widespread drug resistance, with 70%–80% of patients experiencing relapse within 2 years (Blagden et al., 2018). Despite advances in contemporary medical technology, the 5-year survival rate for OC remains lower than 50% (Yang et al., 2022). Therefore, new biomarkers are urgently needed to predict and improve the prognosis of patients with OC.

Immunogenic cell death (ICD) is a unique form of regulatory cell death that can participate in immunity, as well activate fitness in immunocompetent hosts through the release of damage-associated molecular patterns (DAMPs) and cytokine immunity and establish long-term immune memory, which is critical for eradicating pathogens and balancing antitumor immunity to affect the tumor immune cycle (Galluzzi et al., 2020). Two anticancer drugs based on ICD have been developed. One is belantamab mafodotin, which was approved by the FDA in 2020 for the treatment of adult patients with relapsed or refractory multiple myeloma; this drug induces ICD *in vitro* and may contribute to T cell-mediated antitumor responses (Tzogani et al., 2021). The other is lurbinectedin, which has been approved by the FDA for the treatment of small cell lung cancer (Markham, 2020). Multiple ongoing clinical trials have shown that after ICD-inducing chemotherapy, tumors tend to transition from “cold” tumors that respond poorly to immunotherapy to “hot” tumors that respond well to immune checkpoint inhibitors (Jia et al., 2020). The study of these ICD-based therapies undoubtedly provides a new direction for the study of the immunotherapy of “cold” tumors, such as OC. However, the role of ICD-related genes in OC prognosis is still largely unknown.

In this study, we downloaded the gene expression profiles and related clinical information of OC from The Cancer Genome Atlas (TCGA) and Gene Expression Omnibus (GEO) database and collected ICD-related genes from the Genecards database. OC prognosis-related genes were screened from the TCGA cohort and intersected with the ICD-related gene set to obtain ICD-related prognostic genes. Functional enrichment, genetic mutation, and immune infiltration analyses were further carried out to identify underlying mechanisms. Subsequently, through Cox and LASSO regression analyses, we developed a TCGA cohort-based prognostic risk model that included a nine-gene signature. At the same time, we performed external validation with the GEO cohort. Subsequently, we constructed a nomogram by integrating clinicopathological data and prognostic gene signatures to predict patient survival. Finally, we analyzed the functional and immunological differences between high- and low-risk subgroups depending on the above-mentioned risk groups.

## 2 Materials and methods

### 2.1 Data collection and preprocessing

The RNA expression matrix, related clinical information, and RNA expression data from the clinical and follow-up information on OC, cervical cancer, endometrial cancer, and breast cancer were downloaded from TCGA database (https://portal.gdc.cancer.gov/projects/TCGA-OV/). RNAseq data were converted from fragments per kilobase per million format into the transcripts per million (TPM) format and subsequently log2 (TPM + 1) transformed to shrink the numeric range of the data for further analysis. At the same time, samples with incomplete survival data were ruled out. By using the R “survival” package, the expression data were grouped by the median, and overall survival (OS)-related prognostic molecules were screened out through COX regression analysis. The obtained data were considered significant when *p* < 0.01 was satisfied. The Genecards Database (https://www.genecards.org/) is a comprehensive database that integrates genomic, transcriptomic, proteomics, genetic, clinical, and functional information and other resources and is freely available to users (Fishilevich et al., 2017). It was used to retrieve and download ICD-related genes by applying the keyword “immunogenic cell death.” Then, the relevance score provided by the database was utilized to screen out molecules for further research. In this work, the relevance score was computed by factoring in the importance of the different resources associating the gene with the disease (Safran et al., 2021). The median relevance score was set as the threshold to screen out the related genes with strong correlation. OC prognosis-related molecules and ICD-related genes were intersected to obtain the OC ICD-related prognostic gene set, which was used to construct a prognostic model. Finally, two datasets were downloaded from the GEO database (GSE26712 https://www.ncbi.nlm.nih.gov/geo/query/acc.cgi?acc=GSE26712, GSE32062 https://www.ncbi.nlm. nih. gov/geo/query/acc.cgi?acc = GSE32062) for external validation.

### 2.2 Gene expression analysis

Normalized mRNA expression data that had been uniformly processed through the Toil (Vivian et al., 2017) processes of the TCGA-OV cohort and GTEx were downloaded from the UCSC Xena browser (https://xenabrowser.net/datapages/), which was used to compare the expression of prognostic-relevant gene sets between tumor samples and normal samples. Before analysis and comparison, duplicate samples were removed, and the RNAseq data in TPM format were log2 transformed. Finally, the R “ggplot2” package was used for visualization.

### 2.3 Functional enrichment analysis

The STRING database (Szklarczyk et al., 2021) (https://string-db.org/) aims to integrate all known and predicted physical interactions and functional associations between proteins. We used this database to obtain the protein–protein interaction network (PPI) of ICD-related genes. We further organized the PPI network lattice map by using the R “igraph” package. Next, the R “clusterProfiler” package was used to perform Gene Ontology (GO) and Kyoto Encyclopedia of Genes and Genomes (KEGG) enrichment analysis to obtain a deepened understanding of the functional roles of the ICD-genes.

### 2.4 Genetic mutation and immune correlation analysis

The cBioPortal database (Gao et al., 2013) (http://www.cbioportal.org/) is a web-based repository for exploring, visualizing, and analyzing multidimensional cancer genomics data. We used this website to examine the genetic mutation status of 22 ICD-related genes in the TCGA cohort. Subsequently, we applied the ssGSEA algorithm built into the GSVA package to calculate the degree of infiltration of 24 types of immune cells (Bindea et al., 2013), including activated DC (aDC); B cells, CD8 T cells, cytotoxic cells, DC, eosinophils, immature DC (iDC), macrophages, mast cells, neutrophils, NK CD56bright cells, NK CD56dim cells, NK cells, plasmacytoid DC (pDC), T cells, T helper cells, T central memory (Tcm), T effector memory (Tem) cells, T follicular helper (Tfh) cells, T gamma delta (Tgd) cells, Th1 cells, Th17 cells, Th2 cells, and Treg cells.

### 2.5 Construction and validation of a prognostic ICD-Related gene signature

First, a univariate Cox regression model was utilized to evaluate the interaction between ICD-related genes and the OS of OC in the TCGA cohort. ICD-related genes with *p* < 0.05 were identified as factors with potential prognostic value. Subsequently, Lasso regression analysis based on the R “glmnet” package was applied to further screen variables. Ten-fold cross-validation was used to identify the optimal value of λ. Subsequently, genes screened by Lasso regression analysis were input into the multivariate Cox regression model, and the risk score of the ICD-related prognostic model was determined in accordance with the multivariate regression coefficient. Next, the samples were divided into the high-risk and low-risk subgroups on the basis of the median of the risk score as a cutoff. The risk score distribution, survival status, and heatmaps of characteristic ICD-related gene expression were also plotted. The OS Kaplan–Meier curve between the two subgroups was drawn by using the R “survival” package, and the log-rank test was performed. The receiver operating characteristic (ROC) curves of the 3- and 5-year survival rates between the two subgroups were analyzed by utilizing the R “timeROC” package to evaluate the predictive ability of the prediction model with the ICD-related gene signature. Finally, we also analyzed and plotted the progression-free interval (PFI) and disease-specific survival (DSS) Kaplan–Meier curves to estimate the robustness of the prognostic model that we constructed. At the same time, external validation was performed on the two GEO cohorts GSE26712 and GSE32062. Finally, sample validation was performed with three other common female malignancies.

### 2.6 Establishment of the prognostic nomogram

The associations of relevant clinicopathological variables (age, stage, and residual tumor) and ICD-related risk score with OS were measured by using univariate and multivariate Cox proportional hazards regression models. Time-dependent ROC curves were further analyzed and plotted to evaluate the prognostic value of this nomogram. Furthermore, we drew calibration curves by using the R “rms” package and the R “survival” package to assess the agreement between actual and nomogram-predicted survival probabilities.

### 2.7 Functional and immunological analysis of ICD-Related prognostic signature

We performed gene set enrichment analysis (GSEA) on differentially expressed genes between high-risk and low-risk subgroups by using the “clusterProfiler” R package (Subramanian et al., 2005), which used the genome “c2. cp.kegg.v6.2. symbols.gmt” as the reference, to further investigate the underlying functional mechanisms of ICD-related prognostic features. A gene was considered to be significantly enriched when it satisfied false discovery rate <0.25 and P. adjust <0.05. Subsequently, we compared the difference in immune-infiltrating cell scores between the high- and low-risk subgroups to further clarify the correlation of prognostic models with immune status.

### 2.8 Statistical analysis

All statistical analyses in this work were performed by using R software (v3.6.3, https://www.r-project.org/) and corresponding software packages. We used the Wilcoxon rank sum test to compare differences between groups. Spearman’s rank correlation coefficients were calculated to determine the correlation between variables. Kaplan–Meier analysis using the log-rank test was used to assess survival between different subgroups. *p* < 0.05 was considered statistically significant unless stated otherwise.

## 3 Results

**FIGURE 1:**
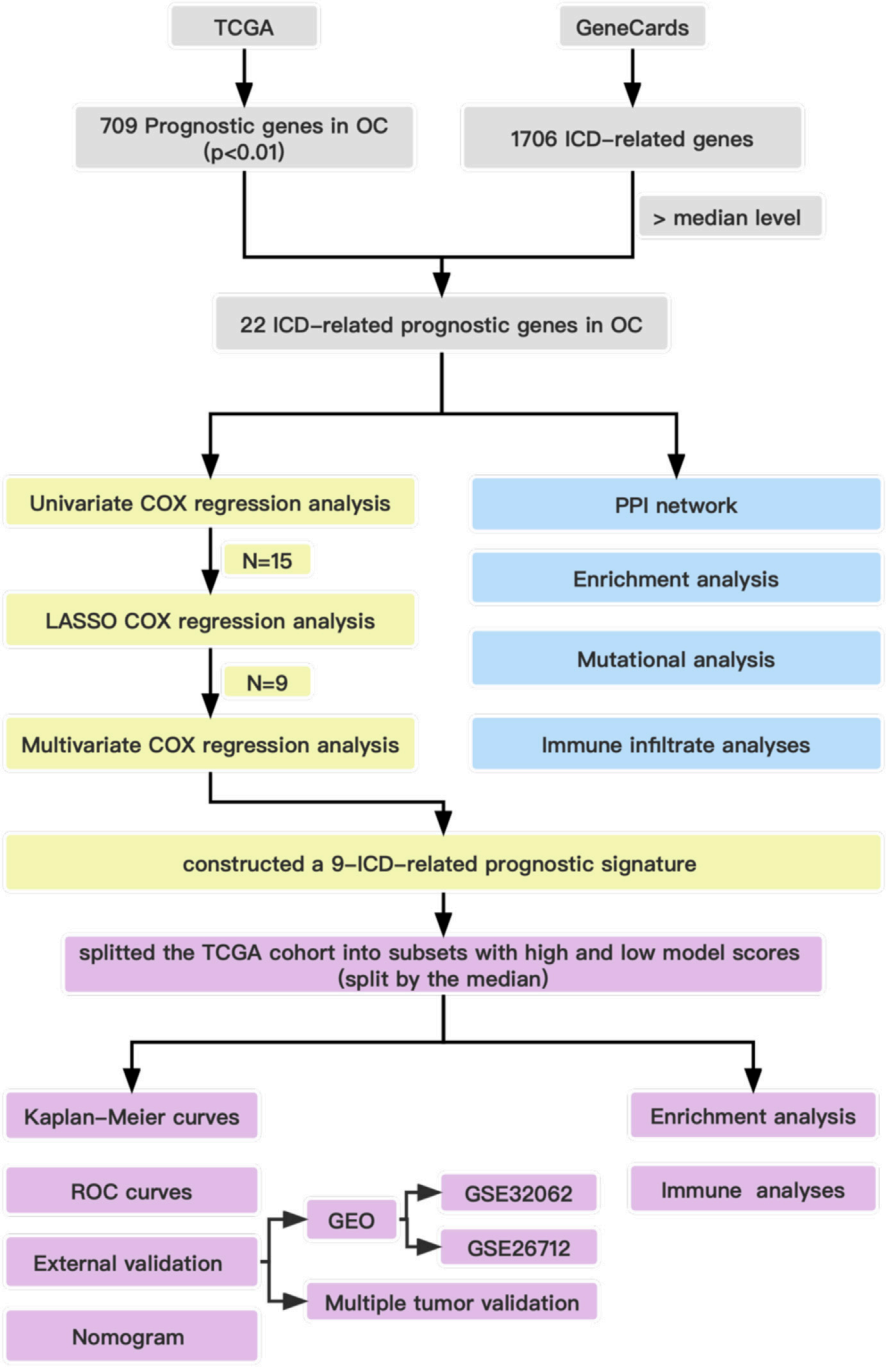
Overall flow chart of the study.

Before presenting the results, we provide the overall flow chart of this work in Figure 1 to help readers understand our work. The baseline clinical characteristics of the patients with OC in this study are summarized in Table 1. We enrolled 374 patients with OC from TCGA as the derivation cohort and 445 patients with OC from GEO as the validation cohort, which included 185 patients in the GSE26712 cohort and 260 patients in the GSE32062 cohort.

**TABLE 1:** Baseline clinical characteristics of the patients with OC in this study.

Characteristic	TCGA	GSE26712	GSE32062	p
N	374	185	260	
Age, median (IQR)	59 (51, 68)	—	—	
Clinical stage, n (%)				<0.001
I	1 (0.2%)	—	0 (0%)	
II	22 (3.5%)	—	0 (0%)	
III	291 (46.1%)	—	204 (32.3%)	
IV	57 (9%)	—	56 (8.9%)	
Histological grade, n (%)				
1	1 (0.2%)	—	0 (0%)	
2	42 (6.6%)	—	131 (20.7%)	
3	319 (50.5%)	—	129 (20.4%)	
4	1 (0.2%)	—	0 (0%)	
B	3 (0.5%)	—	0 (0%)	
X	6 (0.9%)	—	0 (0%)	
OS time, median (IQR)	1028 (519.5, 1661)	1164.35 (660.65, 1879.75)	1245 (810, 1710)	0.001
PFI Time, median (IQR)	444.5 (253, 793.5)	—	—	
DSS Time, median (IQR)	1028 (519.5, 1661)	—	—	

### 3.1 Identification of prognostic genes associated with ICD

**FIGURE 2:**
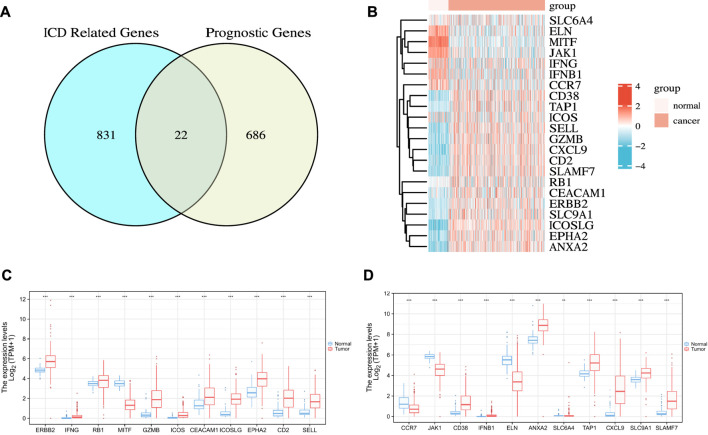
Identification of the candidate ICD-related prognostic genes in the TCGA cohort. **(A)** Venn chart showing the number of OC prognostic genes associated with ICD-related genes. **(B)** Heatmap of the expression of 22 overlapping genes. **(C,D)** Box plot of the expression levels of 22 overlapping genes in OC and normal ovarian tissue. *p* values are *p* ≤ 0.5 (*), *p* ≤ 0.01 (**), *p* ≤ 0.001 (***).

First, we used the “survival” R package to find 709 genes that were significantly associated with OC prognosis in the TCGA cohort (*p* < 0.01) (Supplementary Table S1). Subsequently, we searched the GeneCards database for genes related to ICD by using “immunogenic cell death” as the search term. As a result, we obtained 1706 related genes (Supplementary Table S2), and further used the median relevance score as the threshold to screen out 853 genes. Finally, two gene sets were intersected to obtain 22 ICD-related OC prognostic genes, as shown in Figure 2A. These genes included 10 risk factors (hazards ratio [HR] > 1), namely, ERBB2, RB1, MITF, ICOSLG, EPHA2, JAK1, ELN, ANXA2, SLC6A4, and SLC9A1, and 12 protective factors (HR < 1), namely, IFNG, GZMB, ICOS, CEACAM1, CD2, SELL, CCR7, CD38, IFNB1, TAP1, CXCL9, and SLAMF7.

Next, we analyzed the differences in the expression profiles of these 22 genes in OC samples from TCGA and the corresponding normal tissue samples from GTEx then drew a correlation heat map, as shown in Figure 2B. The results showed that ERBB2, IFNG, RB1, GZMB, ICOS, CEACAM1, ICOSLG, EPHA2, CD2, SELL, CD38, IFNB1, ANXA2, TAP1, CXCL9, SLC9A1, and SLAMF7 were highly expressed in tumor tissues. By contrast, MITF, CCR7, JAK1, ELN, and SLC6A4 were lowly expressed in tumor tissues. The differences were all statistically significant (Figures 2C,D).

### 3.2 Biological function analysis of ICD-Related prognostic genes

**FIGURE 3:**
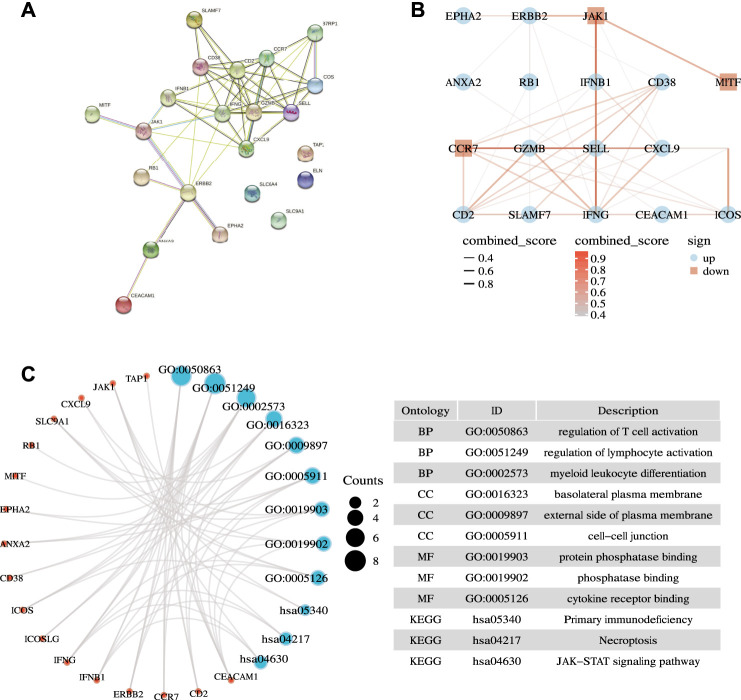
Biological function analysis. **(A)** Interactions among candidate genes shown by the PPI network obtained by using the STRING database. **(B)** Correlation network of selected candidate genes (different color depths represent the strength of the correlation coefficient. Red represents down-regulation, and blue represents up-regulation). **(C)** Results of GO and KEGG analyses.

We first applied the STRING database to analyze the PPI network of the 22 ICD-related prognostic genes. We set the parameter to “*Homo sapiens*” with medium confidence (0.400). We obtained enrichment *p* < 1.0e −16 (Figure 3A). We further drew PPI network dot plots showing high and low expression levels and combined scores (Figure 3B). Subsequently, we performed GO and KEGG enrichment analysis on the genes. Under the conditions of P. adj <0.05 and q value <0.2, we identified 216 BPs, 15 CCs, 5 MFs, and 18 KEGGs (Supplementary Table S3). GO enrichment analysis revealed that ICD-related prognostic genes were significantly enriched in a variety of immune-related biological processes. KEGG pathway analysis demonstrated that the enriched pathways of these cancer-related genes were mainly necroptosis, JAK−STAT signaling pathway, and natural killer cell-mediated cytotoxicity. We visualized three representatives of each item, and the results are presented in Figure 3C.

### 3.3 Mutation and immunological analysis of ICD-Related prognostic genes

**FIGURE 4:**
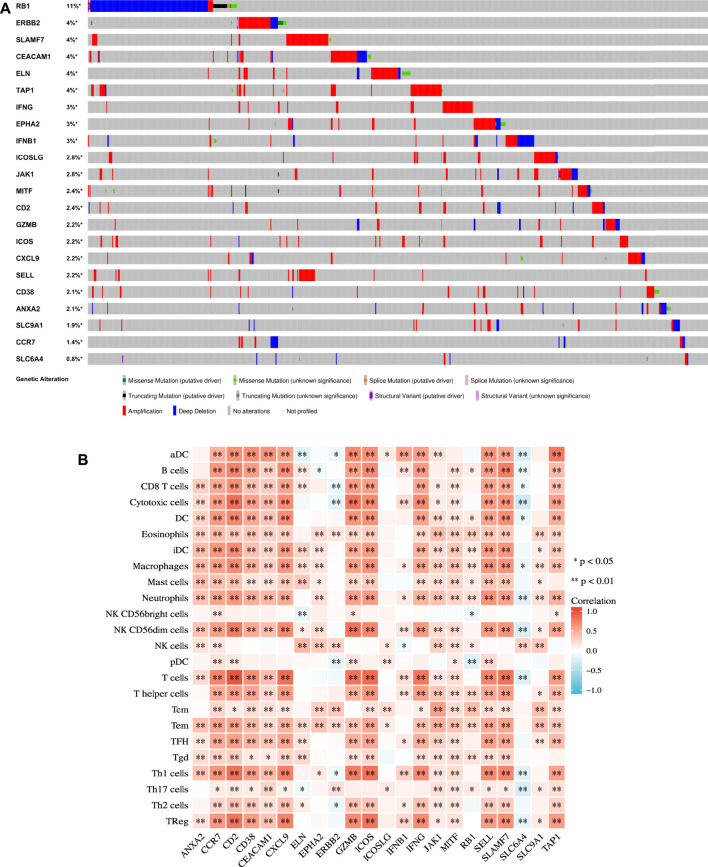
Mutation and immunological analysis. **(A)** Genetic mutation status of 22 genes in the TCGA cohort in the cBioPortal website. **(B)** Correlation between 22 ICD-related prognostic genes and 24 types of immune cells calculated by the ssGSEA algorithm. *p* values are *p* ≤ 0.5 (*), *p* ≤ 0.01 (**), *p* ≤ 0.001 (***).

The extent of inherited mutations partly explains the role of genes in disease progression. We searched the cBioPortal website for genetic mutations in the 22 genes (Figure 4A). We discovered that among the genes, RB1 had the highest mutation frequency, accounting for 11% of mutations, and its main mutation type was deep deletion. Five genes had the mutation frequency of 4%. They included ERBB2, SLAMF7, CEACAM1, ELN, and TAP1, and their mutation form was mainly amplification.

We applied the ssGSEA method to calculate the correlation between each factor and the 24 types of immune cells (Figure 4B) to further evaluate the relationship between the 22 ICD-related prognostic genes and immune cell infiltration. Our results demonstrated that EPHA2, ERBB2, ICOSLG, and SLC9A1 were weakly associated with immune cells. In addition, except for SLC6A4, most of the other genes were positively correlated with immune cells. These findings echoed the results of the functional enrichment analysis discussed above.

### 3.4 Construction of ICD-Related prognostic signature in the TCGA cohort

**FIGURE 5:**
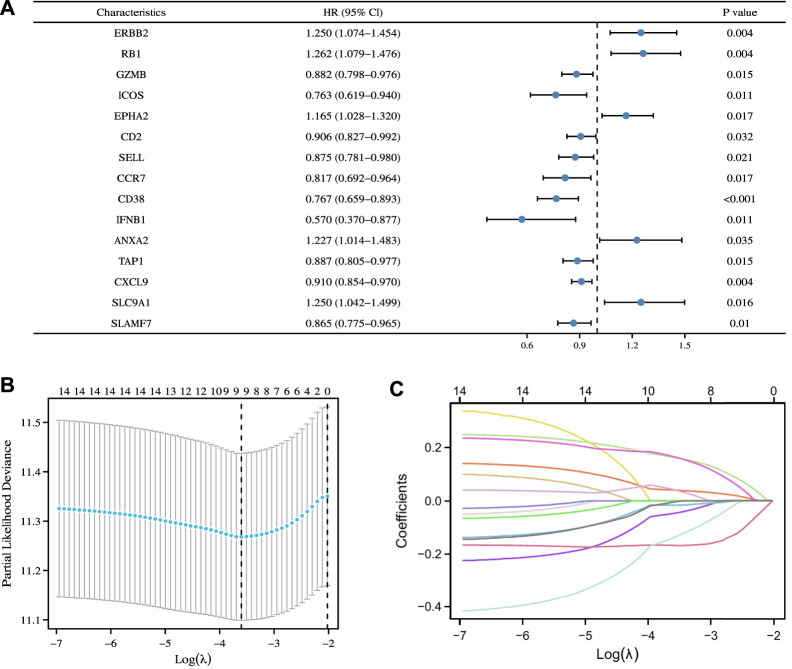
Construction of ICD-related prognostic signature in the TCGA Cohort. **(A)** Univariate Cox regression analysis identified 15 ICD-related genes associated with OS in patients with OC. **(B)** LASSO Cox regression analysis determined 15 ICD-related genes as the optimal combination for the ICD-related prognostic signature construction. **(C)** Plot of variable trajectories generated for log(λ) sequences in LASSO Cox regression analysis.

First, we conducted univariate Cox regression analysis on the above 22 ICD-related genes, and our results revealed that 15 genes were significantly associated with the prognosis of patients with OC (Figure 5A). Next, we included these prognostic genes in further LASSO Cox regression analysis. Nine characteristic ICD-related prognostic genes were identified on the basis of the base penalty parameter (λ) (Figures 5B,C). These genes included ERBB2, RB1, CCR7, CD38, IFNB1, ANXA2, CXCL9, SLC9A1, and SLAMF7.

**FIGURE 6:**
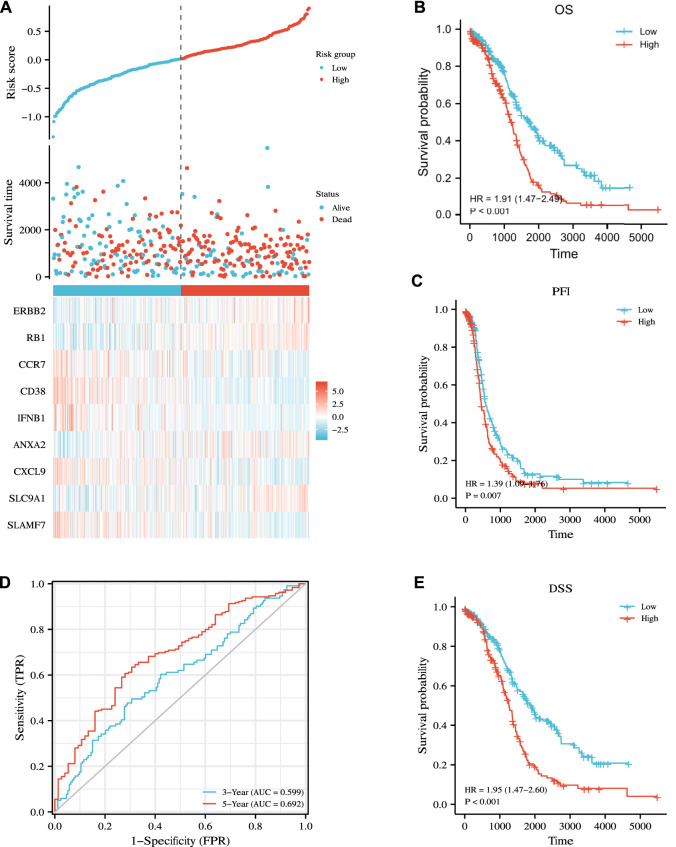
Prognostic analysis of the nine-gene signature model in the TCGA cohort. **(A)** Distribution of risk score, OS, survival status (blue dots indicate alive, red dots indicate death) and the expression heatmaps of nine genes. **(B,C,E)** Kaplan–Meier analysis of OS, PFI, DSS curves in the low- or high-risk subgroups of patients with OC. **(D)** AUC of time-dependent ROC curves for the prediction of 3- and 5-year OS in TCGA.

On this basis, we calculated the risk score of our new prognostic model through multivariate Cox regression analysis by using the following formula: risk score = 0.0521608 × ERBB2 expression +0.20999961 × RB1 expression + (−0.0850947) × CCR7 expression + (−0.1610273) × CD38 expression + (−0.2437315) × IFNB1 expression +0.09886907 × ANXA2 expression + (−0.0133657) × CXCL9 expression +0.22922048 × SLC9A1 expression + (−0.0492765) × SLAMF7 expression + (−2.427119). Finally, we explicitly separated the patients in the TCGA cohort into the high- and low-risk subgroups on the basis of their intermediate survival scores. Risk factor maps, including risk score distributions, the survival status of patients, risk subgroups, and the heatmap of risk gene expression in the two cohorts were also drawn. As shown in Figure 6A, the high-risk subgroup had significantly more deaths than the low-risk subgroup. Similarly, Kaplan–Meier curve analysis illustrated that patients in the high-risk subgroup had a significantly poorer OS than those in the low-risk subgroup (*p* < 0.001, Figure 6B). Time-dependent ROC analysis was utilized to further assess OS predictive power (Figure 6D), yielding the AUC values of 0.599 and 0.692 for the prediction of OS at 3 and 5 years, respectively. We also predicted survival differences in PFI and DSS between the two subgroups to demonstrate the robustness of our predictive model. Our results showed that the high-risk subgroup had worse PFI and DSS outcomes than the low-risk subgroup with *p* = 0.007 and *p* < 0.001, respectively (Figures 6C,E).

### 3.5 Validation of the ICD-Related prognostic signature in the GEO cohort

**FIGURE 7:**
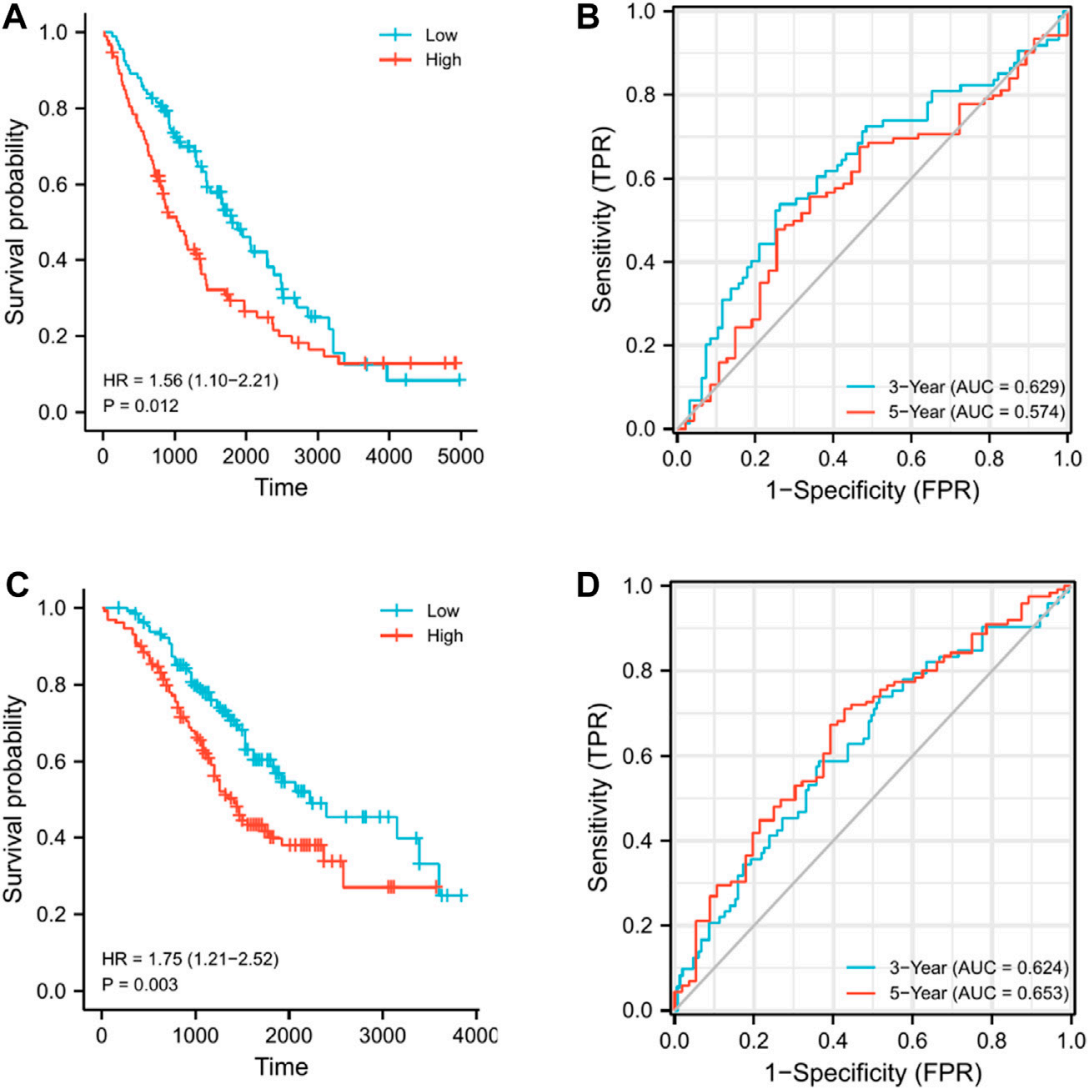
Validation of the nine-gene signature model in GEO cohorts. **(A)** Kaplan–Meier curves for the OS of patients in GSE26712, which was divided into high- and low-risk groups. **(B)** AUC of time-dependent ROC curves for predicting 3- and 5-year OS in GSE26712. **(C)** Kaplan–Meier curves for the OS of patients in GSE32062, which was divided into high- and low-risk groups. **(D)** AUC of time-dependent ROC curves for predicting the 3- and 5-year OS in GSE32062.

We assessed the reproducibility of the prognostic signature in two independent GEO cohorts. (GSE26712 and GSE32062) to validate our findings in the TCGA cohort. Patients in both cohorts were divided into the high- and low-risk subgroups in accordance with the median risk score derived from the risk score formula above, and survival and time-dependent ROC curve analyses were performed on OS. Kaplan–Meier analysis showed that in both cohorts, the high-risk group had significantly worse OS than the low-risk group (*p* = 0.012 and *p* = 0.003) (Figures 7A,C). In addition, the results of time-dependent ROC curve analysis revealed that the AUCs for the 3- and 5-year OS in the GSE26712 cohort were 0.629 and 0.574, respectively (Figure 7B), and were 0.624 and 0.653 in the GSE32062 cohort, respectively (Figure 7D). These results all verify the robustness of the prognostic model that we constructed.

### 3.6 Validation of the ICD-Related prognostic signature in other common female malignancies

We also performed survival analysis and time-dependent ROC curve analysis by using the data of three common female malignant tumors (cervical, endometrial, and breast cancers) in TCGA to further verify the universality of the prognostic signature that we constructed. Kaplan–Meier analysis showed that OS, PFI, and DSS in the high-risk group were significantly worse than those in the low-risk group. (*p* = 0.014, 0.005, and 0.024) (Supplementary Figures S1B,C,E). In addition, the results of time-dependent ROC curve analysis revealed that our constructed prognostic signature can well predict the survival time of cervical cancer (Supplementary Figure S1D). The AUC at 5 years, which had the value of 0.656, was the highest. However, the prediction signature that we constructed did not demonstrate obvious prognostic ability for the data of endometrial cancer (Supplementary Figure S2). Finally, for breast cancer (Supplementary Figure S3), the OS of the high-risk group was significantly worse (*p* = 0.032) than that of the low-risk group. However, no significant difference was found for PFI and DSS. Meanwhile, the results of the time-dependent ROC curve showed that the predictive ability of the ICD-related gene signature for breast cancer was not ideal.

### 3.7 Establishment of a reliable nomogram for the prediction of OC prognosis

**FIGURE 8:**
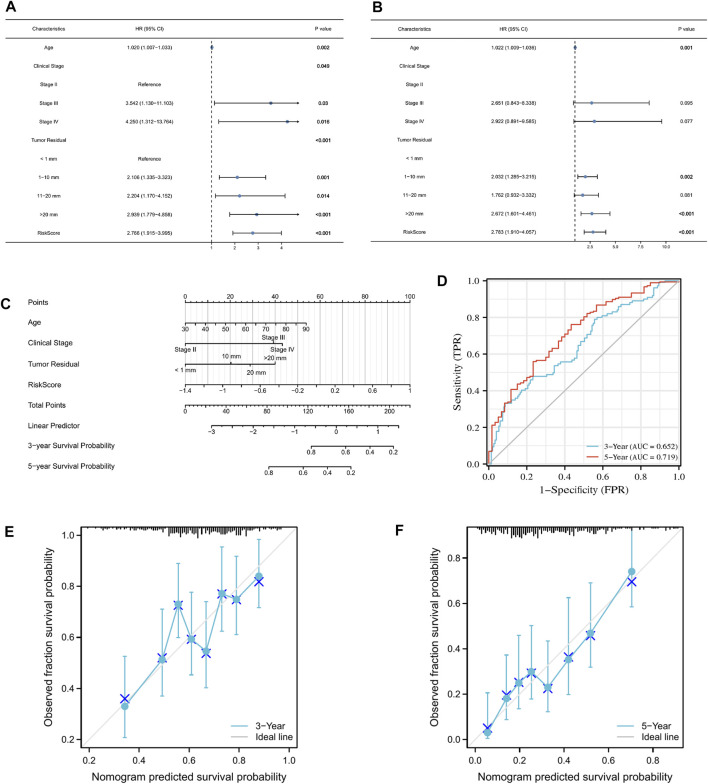
Construction of a nomogram that accurately predicts OC prognosis. **(A,B)** Univariate and multivariate Cox regression model incorporating clinicopathological factors and risk score. **(C)** Nomogram model based on risk model and clinical features. **(D)** Predictive efficacy of the nomogram for 3- and 5-year survival verified through ROC curves. **(E,F)** Calibration plots showing the association of the predicted 3- and 5-year OS with actual survival duration.

Age, FIGO stage, residual tumor, and risk score were included in the univariate and multivariate Cox regression models. The univariate cox regression results showed that age, FIGO stage, residual tumor, and risk score were all significantly correlated with OS (Figure 8A). Multivariate Cox regression analysis revealed that age, residual tumor of 1–10 mm, residual tumor >20 mm, and risk score did appear to be independent prognostic factors for the OS of patients with OC (Figure 8B). Next, we incorporated these clinicopathological parameters and the risk score to construct a nomogram to evaluate survival outcome (Figure 8C), with high total points indicating the worsened prognosis of the patient. Time-dependent ROC curves were further plotted to confirm that the nomogram was highly powerful in predicting patient survival outcomes (Figure 8D). In addition, the calibration curve plotting the predicted probability of survival was generated. As shown in Figures 8E,F, the calibration curve and C index (Concordance = 0.659 [se = 0.023]) indicated that the prediction results of the nomogram had good fit.

### 3.8 Functional enrichment analysis of the ICD-Related prognostic signature and interaction with immune cell infiltration

**FIGURE 9:**
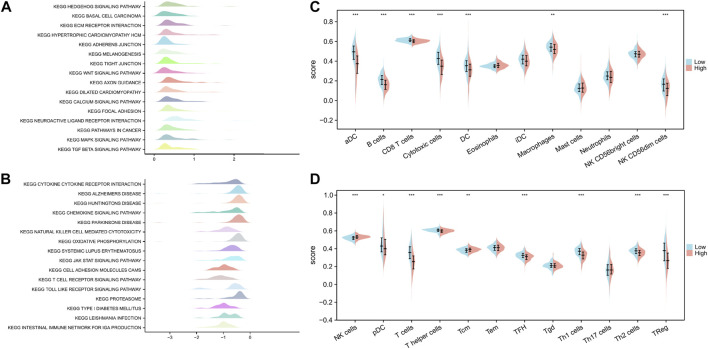
Functional enrichment analysis of ICD-related prognostic signature and interaction with immune cell infiltration. **(A,B)** GSEA revealed biological differences between high- and low-risk subgroups. **(C,D)** Differences in the scores of 24 immune-infiltrating cells between high-risk and low-risk subgroups. *p* values are *p* ≤ 0.5 (*), *p* ≤ 0.01 (**), *p* ≤ 0.001 (***).

We performed GSEA to characterize the biological differences between high- and low-risk subgroups as shown in Figures 9A,B. Cytokine–cytokine receptor interaction, chemokine signaling pathway, NK cell-mediated cytotoxicity, oxidative phosphorylation, systemic lupus erythematosus, JAK–STAT signaling pathway, cell adhesion molecule cams, T cell receptor signaling pathway, Toll-like receptor (TLR) signaling pathway, proteasome, and primary immunodeficiency were enriched in the low-risk subgroups. At the same time, tumorigenic pathways, such as hedgehog signaling pathway, ECM receptor interaction, WNT signaling pathway, calcium signaling pathway, pathways in cancer, MAPK signaling pathway, and TGF beta signaling pathway, were significantly activated in the high-risk subgroup, suggesting that the high-risk subgroup had a detrimental effect on survival outcomes.

We quantified the differences in the scores of 24 immune-infiltrating cells between the high- and low-risk subgroups to further explore the correlation between our constructed genetic signature risk score and immune status. Wilcoxon rank sum test showed that only NK cells and Tcm were enriched in the high-risk subgroup. However, most immune-infiltrating cells were significantly enriched in the low-risk subgroup. These cells included pDC, T cells, T helper cells, TFH, Th1 cells, Th2 cells, Treg cells, aDC, B cells, CD8 T cells, cytotoxic cells, DC, macrophages, and NK CD56dim cells (Figure 9C,D).

## 4 Discussion

OC, as the main cause of death by gynecological malignancies, is a perennial object of interest of researchers. New treatment options, such as targeted therapy, biological therapy, and immunotherapy, have been introduced, but their results continue to be unsatisfactory. In recent years, immune checkpoint blockers have been recognized as the most promising method for cancer treatment. However, in a variety of immunologically “cold” tumor types, including OC, their therapeutic efficacy is largely limited by factors, such as the lack of tumor antigens, the activation of T cells, priming, and infiltration (Bonaventura et al., 2019). Recently, the advent of ICD has heralded a new dawn in the diagnosis and treatment of OC. Studies have clearly pointed out that ICD-based cancer vaccines can be immunogenic against “cold” tumors while increasing sensitivity to immunotherapy (Jin and Wang, 2021). Therefore, understanding the intimate relationship between differentially expressed genes in OC and ICD will allow us to explore the therapeutic potential of ICD-based treatments on a deep level.

We performed the first comprehensive identification and investigation of the ICD-related prognostic signature of OC. Our work can expand the ideas for the improvement of OC prognostic prediction and the guidance of individualized treatment. In this study, we mined 22 ICD-related genes with distinct prognostic implications in OC. We applied unpaired samples to further analyze the expression of these 22 genes in OC tissue in TCGA and the corresponding normal tissue in GTEx. We found that only five genes (MITF, CCR7, JAK1, ELN, and SLC6A4) functioned as tumor suppressors, whereas the remaining 17 genes functioned as oncogenes. Next, we performed further functional analysis on these genes. As a result, we discovered that the vast majority were involved in immune-related biological processes. This finding was consistent with the characteristics of ICD. Our KEGG enrichment pathway analysis results showed that ICD-related genes were involved in the JAK–STAT signaling pathway and natural killer cell-mediated cytotoxicity. Previous studies have demonstrated that the ICD-related gene IFN can induce a variety of cell phenotypes by activating the JAK–STAT signaling pathway (Medrano et al., 2017). Minute et al. co-cultured tumor cells and cytotoxic immune cells (such as T lymphocytes and NK cells) and observed the presence of ICD markers. They found that cytotoxic immune cells could induce the release of DAMPs, further triggering the antitumor immune response (Minute et al., 2020). Consistent with our enrichment analysis results, this phenomenon suggested that cytotoxicity is a type of ICD.

The immune system is our primary defense mechanism against exogenous and endogenous threats (Lakins et al., 2018). It mainly includes innate immune cells (such as macrophages, dendritic cells, and natural killer cells) and adaptive immune cells (such as T and B cells) (Chu et al., 2022). Numerous studies have demonstrated that genetic mutations in genes can affect tumor immune status (Xu et al., 2014). Mutations in U2AF1 have been shown to activate innate immune pathways in myeloid malignancies (Smith et al., 2019). Furthermore, p53 mutations can support immune dysfunction by altering the tumor microenvironment, disrupting innate immunity by modulating the TLR signaling pathway, and promoting immune privilege and the ability to survive by disrupting cell-mediated immunity (Agupitan et al., 2020). Therefore, we analyzed the genetic mutation status of ICD-related genes and their association with immune-infiltrating cells. We found that only RB1 mutations were dominated by deep deletion mutations. By contrast, the mutations of other genes were dominated by amplification. The results of immune infiltration analysis showed that most genes were positively correlated with immune-infiltrating cells. This situation indicated that the prognostic model we constructed may have a certain predictive ability in immunotherapy.

Next, we constructed the nine-gene signature of the prognostic risk model by performing univariate/multivariate Cox regression and LASSO regression analyses. This signature included ERBB2, RB1, CCR7, CD38, IFNB1, ANXA2, CXCL9, SLC9A1, and SLAMF7. ERBB2, commonly referred to as HER2, is a oncogene located on chromosome 17 that encodes a member of the epidermal growth factor receptor family of receptor tyrosine kinases (Li et al., 2022). ERBB2 amplification and mutation have been identified in many cancer types (Abrahao-Machado and Scapulatempo-Neto, 2016; Lin et al., 2021). They promote cancer cell growth and invasion and portend poor prognosis (Uchida et al., 2021). Currently developed nanoparticles targeting ERBB2 can enhance ICD effects at tumor sites (Zheng et al., 2020). CCR7 is a lymphocyte-specific G protein-coupled receptor (Birkenbach et al., 1993) that executes a unique antagonistic role in tumorigenesis by transferring tumor cells to the T cell region of lymph nodes (Zlotnik et al., 2011). It is essential for initiating adaptive immune responses. Interestingly, however, we found that some studies point to the opposite role of CCR7; specifically, CCR7 is induced in some cancer cells and contributes to metastasis formation (Gerken et al., 2022). CD38 is a messenger for intracellular calcium mobilization (Schmid et al., 2011), which participates in and regulates immune cell differentiation, activation, and tolerance (Malavasi et al., 2008). Several studies have shown that the expression of CD38 in tumors can induce proliferation and inhibit apoptosis (March et al., 2007) and participate in processes, such as tumor cell energy metabolism (Liao et al., 2020), and immune tolerance and resistance (Chen et al., 2018). IFNB1 is a cytokine of the well-known signaling protein type I interferon family and is involved in cell differentiation and antitumor defense (Daman and Josefowicz, 2021). It has powerful antiproliferative, proapoptotic, antiangiogenesis, and immunomodulatory functions (Ambjorn et al., 2013). The ANXA2 gene encodes a member of the calcium-dependent phospholipid-binding protein family, which may play a role in regulating cell growth and signal transduction pathways (Bharadwaj et al., 2013). A growing body of evidence shows that the dysregulation of ANXA2 expression is associated with tumorigenesis and immunity in a variety of cancers, such as glioma (Li et al., 2021), oral squamous cell carcinoma (Ma and Wang, 2021), pancreatic cancer (Karabulut et al., 2020), colorectal cancer (Hu et al., 2020), breast cancer (Sharma and Jain, 2020), thyroid cancer (Qin et al., 2020), and gastric cancer (Han et al., 2017). A recent experiment explored the effect of OC cell-derived exosomal ANXA2 on peritoneal implantation and tumor metastasis and its underlying mechanism (Gao et al., 2021). CXCL9, a member of the CXC chemokine subfamily, encodes secreted proteins that participate in immune regulation and inflammatory processes (Balkwill, 2004). Research on its role in tumors remains controversial. It can either act as an oncogenic factor to promote tumor progression or as a tumor suppressor to exert antitumor effects (Xiu and Luo, 2021). Our analysis showed that it may function as the former. SLC9A1 is a member of the solute carrier family 9. It encodes a protein that acts as a plasma membrane transporter with a crucial role in regulating pH homeostasis, cell migration, and cell volume (Cardone et al., 2019). SLAMF7 is a member of the signaling lymphocyte activating molecule family of receptors, which could be involved in adaptive immune responses. (Cannons et al., 2011). Studies have indicated that its expression has prognostic significance in cancers dominated by T cell exhaustion (O'Connell et al., 2021). In addition, a recent study found that SLAMF7 can mediate ICD in colorectal cancer cells (Roh et al., 2021). These genes can promote or inhibit tumor immune pathways through various mechanisms. However, whether they play an important role in the prognosis of patients with OC by affecting ICD remains unclear.

In recent years, on the basis of the characteristics of ICD, researchers have explored immunotherapy based on ICD in an attempt to overcome the limitations of conventional tumor treatment. However, the potential relationship between ICD and OC prognosis has yet to be elucidated. Our study divided OC samples from the TCGA database into high- and low-risk subgroups on the basis of the median risk score and assessed their prognostic value for OS, PFI, DSS, and 3-year and 5-year survival ROC curves. Patients in the high-risk subgroup had a significantly worse prognosis than those in the low-risk subgroup. The risk model we constructed had good predictive value for the 3- and 5-year survival rates of patients. In addition, we performed external validation by using two sets of GEO cohorts, further illustrating the power of the prognostic model. We also used the data on cervical, endometrial, and breast cancers in the TCGA database to validate the usefulness of our prognostic signature for multiple cancers, and our results showed that the prognostic signature that we constructed has a certain universality. Subsequently, we constructed a nomogram incorporating relevant clinicopathological factors to predict survival probability. After completing these works, we analyzed the differentially expressed genes between high- and low-risk subgroups then performed GSEA. Interestingly, we found that various cancer-related pathways were enriched in the high-risk subgroup. This situation further explained the poor prognosis of the high-risk subgroup. Enriched pathways in the low-risk subgroup were mainly closely related to the immune response. Studies have also illustrated that most T-cell markers, including CD8^+^ T cells, Th1, and Tem cells, were closely associated with good prognosis (Bindea et al., 2013). Therefore, we further analyzed the differences in the abundance of immune-infiltrating cells between the two groups. In coincidence with previous research findings, our results showed that most immune-infiltrating cells were significantly enriched in the low-risk subgroup. This situation suggested that the risk score formed by our nine-gene signature was inversely associated with immune cell infiltration and that our model may predict immune responses in tumors. Similarly, Wang et al. recently constructed an ICD-related classification signature to predict prognosis and immunotherapy response in head and neck squamous cell carcinoma (Wang et al., 2021). In their prognostic signature, the high-risk cohort score also corresponded to poor OS. In addition, patients with high-risk scores were inversely associated with CD8 T cells. This association was also confirmed in our study. Therefore, we can reasonably speculate that the identification of ICD-related biomarkers may be beneficial for a variety of malignant tumors and that these biomarkers can help identify patients with tumors who can benefit from immunotherapy (Wang et al., 2021).

In summary, we provided additional insights into the association between ICD-related genes and OC prognosis. Our newly constructed ICD signature demonstrated certain sensitivity and specificity as a prognostic predictor of OC. The prognostic nomogram based on the ICD-related signature also showed an excellent ability to forecast the OS of patients with OC. However, our research continues to have some deficiencies. The ICD-related prognostic model that we established was based only on public databases for bioinformatics analysis. In the future, we will perform experimental studies and validate the comprehensive roles of ICD-related genes in the progression of OC.

## Data Availability

The datasets presented in this study can be found in online repositories. The names of the repository/repositories and accession number(s) can be found in the article/Supplementary Material.
