# Supplementary material for: Immunogenic cell death-related gene landscape predicts the overall survival and immune infiltration status of ovarian cancer
Source: Front Genet. 2022 Nov 8;13:1001239. doi: 10.3389/fgene.2022.1001239 (PMC9679378; doi:10.3389/fgene.2022.1001239)
Supplement: Supplementary file 1 [file Table2.DOCX]

**raw data and code**

https://www.jianguoyun.com/c/sd/1593bdf/1a939680168eb835
