# Supplementary material for: Immunogenic cell death-related gene landscape predicts the overall survival and immune infiltration status of ovarian cancer
Source: Front Genet. 2022 Nov 8;13:1001239. doi: 10.3389/fgene.2022.1001239 (PMC9679378; doi:10.3389/fgene.2022.1001239)
Supplement: Supplementary file 2 [file Table3.DOCX]

Supplementary Figure 1 **The prognostic analysis of the 9-gene signature model in** **cervical cancer.**

**(A)**Distribution of risk score, OS, survival status (green dots indicate alive, orange dots indicate death) and the 9 genes expression heatmaps **(B, C, E)** Kaplan-Meier analysis of OS, PFI, DSS curves in low-or high-risk subgroups of cervical cancer patients**(D)** The AUC of time-dependent ROC curves for predicting 1-, 3-, and 5-year OS in TCGA.

Supplementary Figure 2 **The prognostic analysis of the 9-gene signature model in endometrial cancer.**

**(A)**Distribution of risk score, OS, survival status (green dots indicate alive, orange dots indicate death) and the 9 genes expression heatmaps **(B, C, E)** Kaplan-Meier analysis of OS, PFI, DSS curves in low-or high-risk subgroups of endometrial cancer patients**(D)** The AUC of time-dependent ROC curves for predicting 1-, 3-, and 5-year OS in TCGA.

Supplementary Figure 3 **The prognostic analysis of the 9-gene signature model in breast cancer.**

**(A)**Distribution of risk score, OS, survival status (green dots indicate alive, orange dots indicate death) and the 9 genes expression heatmaps **(B, C, E)** Kaplan-Meier analysis of OS, PFI, DSS curves in low-or high-risk subgroups of breast cancer patients**(D)** The AUC of time-dependent ROC curves for predicting 1-, 3-, and 5-year OS in TCGA.
